# Synthetic Comparative Analysis of Cardiovascular Disease Profiles Using Quantum Information Measures in Indian and Global Populations

**DOI:** 10.3390/diagnostics16172878

**Published:** 2026-09-07

**Authors:** Santosh Kumar Sahoo, Sumant Kumar Mohapatra, Srikanta Kumar Mohapatra, Chander Prabha, Jayashree Mohanty, Deepudev Sahadevan, Sumendra Yogarayan, Balamurugan Balusamy

**Affiliations:** 1 School of Computing Science and Engineering, VIT Bhopal University, Sehore 466114, India; santoshkumarsahoo@vitbhopal.ac.in; 2 Department of CSE (AI&ML), School of Engineering, Dayananda Sagar University, Bengaluru 562112, India; sumantkumar-aiml@dsu.edu.in; 3 Chitkara University Institute of Engineering and Technology, Chitkara University, Rajpura 140401, Punjab, India; srikanta.mohapatra@chitkara.edu.in (S.K.M.); chander.prabha@chitkara.edu.in (C.P.); 4 Department of Computer Science and Engineering, Chandigarh University, Mohali 140413, India; jayashree.e15737@cumail.in; 5 Faculty of Mathematics and Data Sciences, Emirates Aviation University, Dubai P.O. Box 53044, United Arab Emirates; deepu.dev@eau.ac.ae; 6 Faculty of Information Science and Technology (FIST), Multimedia University (MMU), Jalan Ayer Keroh Lama, Bukit Beruang, Ayer Keroh 75450, Malaysia; 7 School of Engineering and IT, Manipal Academy of Higher Education, Dubai Campus, Dubai P.O. Box 345050, United Arab Emirates; balamurugan.balusamy@manipaldubai.com

**Keywords:** cardiovascular, quantum, population, hypertension

## Abstract

**Background/Objectives:** Cardiovascular diseases (CVDs) represent the foremost public health challenge of recent days. According to the World Health Organization (WHO), nearly 32% of people die from CVDs each year. This paper introduces a quantum-inspired, information-theoretic framework for comparing CVD risk-factor architectures in modeled Indian and global populations. **Methods:** Each synthetic population is represented as a population state matrix (ρ) built from discretized epidemiological variables (age, sex, smoking, diabetes, hypertension, and dyslipidemia). Systemic uncertainty is quantified with von Neumann entropy (equivalent to Shannon entropy for diagonal states), inter-factor dependence with Systemic Mutual Information (SMI), and synthetic population similarity with Uhlmann fidelity. **Results:** In illustrative modeled scenarios (N=10,000 synthetic profiles per synthetic population), diabetes entropy is higher in the Indian comparator (0.88 vs. 0.61 bits), diabetes–hypertension SMI is larger in India (≈0.46 vs. 0.22 bits), and smoking–hypertension dependence is stronger globally (0.36 vs. 0.21 bits). Overall fidelity is F≈0.76. **Conclusions:** These results are hypothesis-generating point estimates and require clinical data verification on harmonized synthetic individual-level data before clinical or policy use. This study represents a quantum information-theoretic framework for comparing modeled CVD risk profiles. Illustrative results suggest stronger diabetes–hypertension dependence in the Indian scenario and stronger smoking–hypertension dependence in the global comparator.

## 1. Introduction

Cardiovascular diseases (CVDs) are established public-health issues when it comes to mortality figures in the world [1]. The World Health Organization believes that CVDs are responsible for approximately 18 million deaths annually, representing over 32 percent of the total number of deaths on the entire globe. The financial implications are just as severe, with healthcare costs and decreased productivity amounting to hundreds of billions of dollars per annum.

### 1.1. The South Asian Paradox: A Case for Deeper Analysis

The aspect that makes the problem of CVD very intriguing from a scientific point of view is the unequal distribution of risk factors across certain population groups. In particular, South Asians are at much higher risk, which is termed the “South Asian Paradox” at times [2,3]. Coronary artery disease develops earlier among south Asians by about 10 years compared to their Western counterparts [3]. Also, mortality from this disease is increased, and the underlying pathophysiologic processes seem to be quite different [2,3,4,5]. These individuals are more likely to suffer from the syndrome of insulin resistance, central obesity, and atherogenic dyslipidemia, even if they have a normal body mass index. This is often defined as “thin–fat” syndrome. In addition, there are a number of genetic loci which increase susceptibility to coronary artery disease that occur more frequently. Thus, it becomes clear that risk factor interactions differ among populations.

### 1.2. Limitations of Current Analytical Paradigms

The Framingham Risk Score and other similar risk scores have not been developed for such situations [6]. They cannot capture the complex nature of non-linear and synergetic risk associations of a certain population as these involve either linear or logistic regression analysis. Take diabetes and hypertension as an example. The total risk is far greater than the sum of individual risks, while regression analysis assumes them to be mostly additive in nature. Also, when a model is built with one particular population, it would likely fail when applied to a different population due to the lack of generalization or poor transportability [7]. The current generation of machine learning techniques performs well but may be considered as a black box.

### 1.3. A New Approach: Information-Theoretic Systemic Analysis

In contrast, here we adopt a different strategy. Specifically, instead of relying on the mathematics developed by quantum information theory (QIT) because our health data are quantum–mechanical, we make use of QIT because it is an elegant way to mathematically characterize any information system with correlation. In other words, one should think of a community as an information system in which its “state” represents all its risk factors together. We explicitly consider a population state matrix (ρ) that wraps up all the statistical information about a synthetic population into one object, rather than treating risk factors in isolation. Related quantum computing approaches have been explored in bio-applications for various fields [8,9,10,11,12,13,14,15,16,17,18,19,20,21]:

✓ System Entropy, which tells us how diverse or uncertain a population is with respect to a given risk factor.
✓ System Entropy which measures the heterogeneity or ambiguity of a population with respect to a given risk factor.
✓ Systemic Mutual Information (SMI), that measures the coupling strength between any two risk factors, thus mapping the comorbidity landscape.

**Figure 1:**
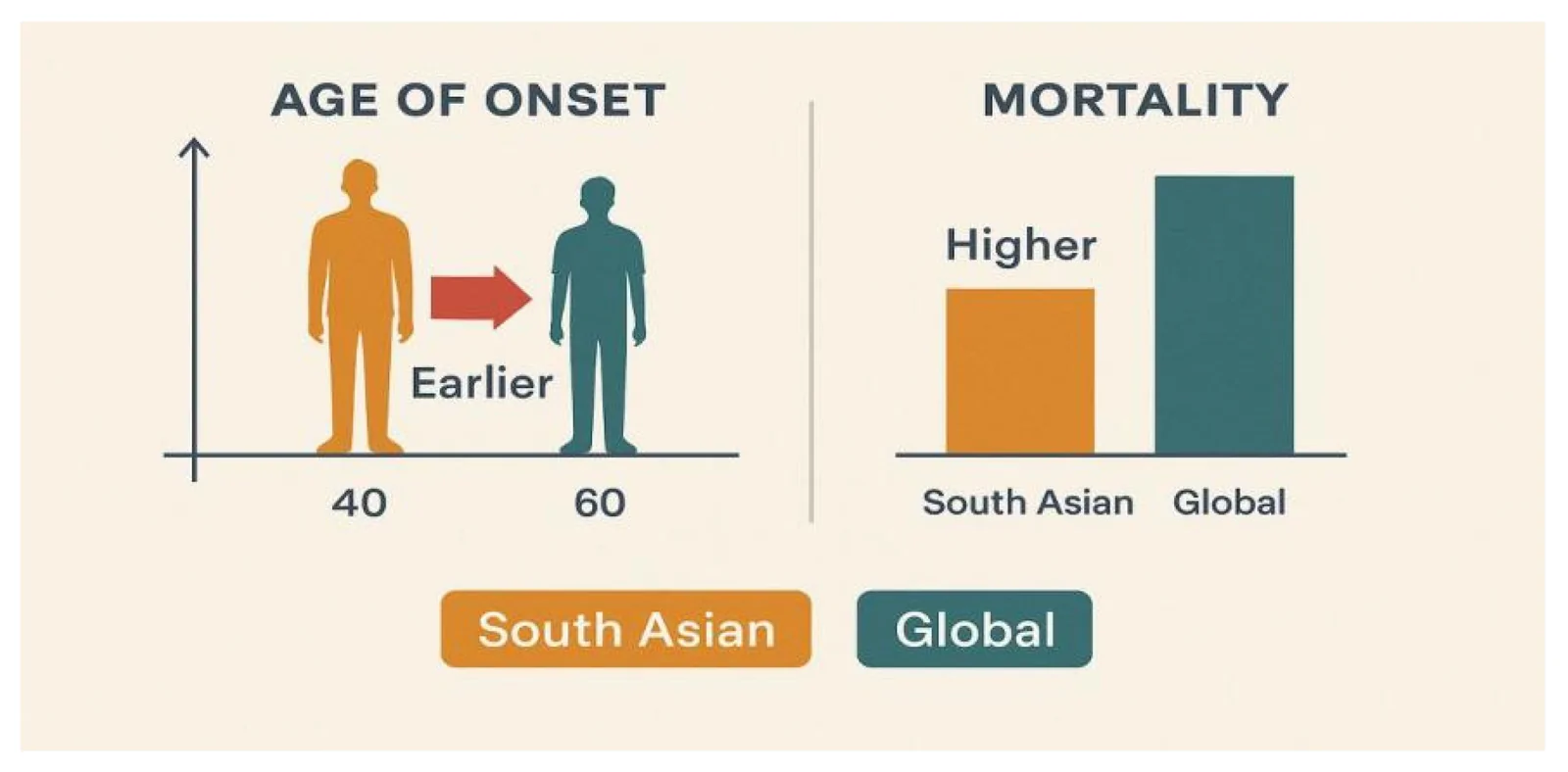
Comparison of key CVD metrics of the South Asian vs. global population paradox.

Figure 1 underscores the earlier age of onset and higher relative mortality that must be considered in a population-specific analysis. The aim is to apply these tools to Indian and global synthetic population that have been modeled and to draw concrete implications for clinical practice and public health policy from the numbers.

## 2. Materials and Methods

[a] Study Design and Data Sources

We performed a comparative in silico analysis based on the published epidemiological data as per link presented in data availability declaration section. Published prevalence patterns and reported risk-factor relationships were used to construct synthetic categorical profiles for two comparison groups. Each modeled synthetic population contains N=10,000 synthetic profiles, large enough to stabilize empirical categorical frequencies while preserving the intended prevalence inputs. The profiles are model-based population scenarios, not directly sampled national synthetic populations. Two synthetic populations were assembled to capture the contrasting disease architectures we sought to study.

India Group: The proposed study for the Indian arm was mainly focused on the data derived from the ICMR-INDIAB study [22], which is a large national survey delivering information on diabetes and metabolic factors in relation at state level. The proposed study would supplement this dataset using information obtained from the Indian arm of the PURE study exploring the interaction between urbanization and lifestyle and cardiovascular outcomes [23]. The combination of both datasets would allow us to address the very high prevalence of metabolic syndrome prevalent among the South Asians. International synthetic population reference is built upon the data available from such publicly accessible databases as NHANES, UK Biobank, and WHO Global Health Observatory database [1,24,25] creating a rough picture of the Western population profile, characterized by low diabetes but with high correlation between CVD and lifestyle factors.

[b] Variable Definition and Discretization

As the proposed framework operates on categorical variables, all continuous measures are discretized into ordered bins before analysis. Table 1 lists the seven risk factors as retained, together with the binning strategy used for each. Seven variables in Table 1 explain a 1024-state categorical space (4×2×2×2×2×4×4), so each population state matrix is a 1024×1024 diagonal density matrix in this classical implementation. The 1024 states are nonuniform and estimated from the synthetic dataset having 10,000 synthetic profiles for each population.

Each synthetic profile maps to one basis state according to selected marginal prevalences and prespecified pairwise dependence targets (e.g., stronger diabetes–hypertension coupling in the Indian scenario and stronger smoking–hypertension coupling in the global comparator). Profiles were sampled from the resulting joint distribution over 1024 states; diagonal entries of ρ are empirical probabilities normalized to unit trace. The parameter Obesity describes the excessive fat content of the body, which leads to different health complications. As per WHO, a higher value (i.e., BMI ≥ 30) of body mass index (BMI) indicates different health issues like blood sugar problems, cardiac issues etc. From the National Family Health Survey 2019–21 in India, the prevalence of obesity was obtained. The below workflows are followed:

(i) Select prevalence and dependence inputs from cited sources; (ii) discretize variables per Table 1; (iii) generate N=10,000 synthetic profiles per synthetic population; (iv) aggregate counts into normalized state probabilities; (v) construct density matrices and marginals by partial trace; (vi) compute entropy, SMI, and fidelity. All numerical estimates are illustrative point estimates without bootstrap confidence intervals, p-values, or sensitivity analysis; clinical data verification and feature analysis is required before clinical conclusions.

### 2.1. Computational and Analytical Pipeline

**Figure 2:**
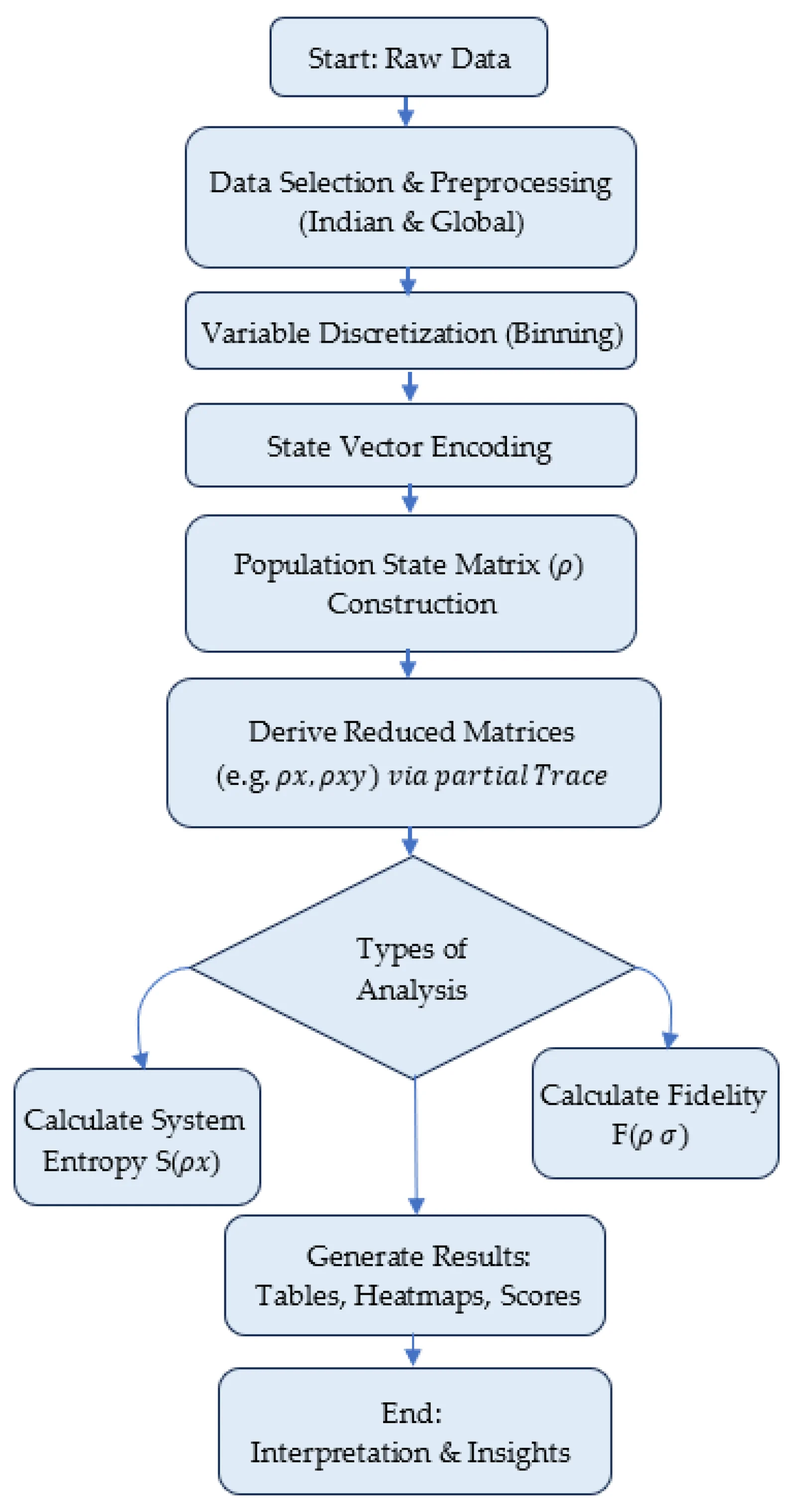
Flowchart of the data processing and analytical pipeline.

The overall workflow is summarized in Figure 2 with N = 10,000 and population state matrix of 1024 × 1024. The epidemiological variables (age, sex, diabetes status, etc.) are preprocessed and binned into the categorical forms described above. Each synthetic individuals’ profile is then encoded as a state vector, and the full set of vectors is aggregated into the population state matrix. This single matrix compactly encodes both the marginal distributions and the joint distributions of all risk factors [22,24,25].

Once the state matrices are in hand, computing the information-theoretic quantities is straightforward. Von Neumann entropy tells us how uncertain each risk factor is at the population level; SMI reveals pairwise dependencies between factors; and fidelity gives us a global similarity score between the two synthetic populations. In the final stage we bring all three measures together to highlight where the Indian and global disease architectures agree and where they diverge.

### 2.2. Population State Matrix Formulation

The starting point of our framework is to turn each synthetic individuals’ clinical profile into a mathematical vector. We take the discretized risk-factor values discussed above (age bin, sex, smoking status, etc.) and assign each combination a basis state in a Hilbert space. The full profile for synthetic individuals k is then the tensor product of these basis states, which we write as a state vector $\left|\Psi_{k}\right\rangle$ [26,27].

*Individual Representation.*

Concretely, for a synthetic individual k whose risk factors have been binned into M categorical variables, the state vector is:

(1) $$\left|\Psi_{k}\right\rangle ={⨂}_{i=1}^{M}\left|x_{i}^{\left(k\right)}\right\rangle ,$$

with $\left|x_{i}^{\left(k\right)}\right\rangle$ being the basis state for the i-th factor (e.g., “diabetic” vs. “non-diabetic,” “smoker” vs. “non-smoker”). The tensor product bundles all of these individual labels into a single vector that lives in the joint space of all risk factors.

*Population Representation.*

One synthetic individual gives us one vector; an entire population of N synthetic individuals give us N vectors. Rather than storing them as a list, we aggregate them into a single object—a density matrix, which we call the population state matrix: [26,27].

(2) $$\rho =\frac{1}{N}\sum_{k=1}^{N}\left|\Psi_{k}\right\rangle \left\langle \Psi_{k}\right|.$$

What makes ρ so useful is that it packs a lot of information into one matrix. Its diagonal entries are just the classical probabilities of seeing particular risk-factor combinations in the synthetic population, while the diagonal entries encode how those factors co-occur. In effect, ρ simultaneously contains every marginal distribution and every joint distribution across all combinations of the six risk factors.

*Reduced Density Matrix via Partial Trace*

Oftentimes we do not want to look at all six risk factors at once—we might be interested in just diabetes, or just the pair diabetes-plus-hypertension. The standard way to “zoom in” is the partial trace: we trace out every variable we are not interested in, and what remains is the reduced density matrix $\rho_{X}$: [26].

(3) $$\rho_{X}={Tr}_{\mathrm{rest}}(\rho )$$

This keeps all the statistical information about X intact while discarding everything else.

*Partial Trace and Reduced Matrices:*

More formally, if we split our system into two subsystems, A and B, we can recover the marginal state of A by summing over all possible states of B. For a joint state $\rho_{AB}$, the reduced density matrix for A is: [26,28]

(4) $$\rho_{A}={Tr}_{B}(\rho_{AB})=\sum_{j}\left(\langle j|⨂I_{A}\right)\rho_{AB}\left(|j\rangle ⨂I_{A}\right)$$

Here {|j⟩} runs over an orthonormal basis for B, and $I_{A}$ is the identity operator on A. The result is the marginal distribution of A, which retains whatever correlations exist among A’s own categories but averages over B.

*System Entropy (von Neumann Entropy)*

To measure how spread out a population is across the categories of a given risk factor, we use the von Neumann entropy of the corresponding density matrix: [26,27,29]

(5) $$S(\rho )=-Tr(\rho {log}_{2}\rho )$$

This is the matrix-valued analog of Shannon entropy. When the state matrix happens to be diagonal—as it is for our classical epidemiological data—the two coincide exactly, a fact we verify formally in Lemma 2.

*Systemic Mutual Information (SMI)*

While entropy describes a single variable, we also need a way to quantify how much two risk factors “know” about each other. That is the role of Systemic Mutual Information (SMI), defined as: [27,28]

(6) $$I\left(A:B\right)=S\left(\rho_{A}\right)+S\left(\rho_{B}\right)-S\left(\rho_{AB}\right)$$

with $\rho_{A}$ and $\rho_{B}$ being the reduced density matrices and $\rho_{AB}$ the joint state. Intuitively, SMI measures the gap between the sum of individual uncertainties and the joint uncertainty. A non-zero gap means the two variables share statistical structure—knowing one reduces your uncertainty about the other. We prove in Theorem 2 that this quantity is always non-negative.

*State Fidelity*

Finally, we need a single number that summarizes how alike (or unlike) two entire populations are. For this we use the Uhlmann fidelity, which compares two density matrices ρ and σ directly [30,31]:

(7) $$F(\rho ,\sigma )={\left(Tr\;\left(\sqrt{\sqrt{\rho }\;\sigma \sqrt{\rho }}\right)\right)}^{2}.$$

Fidelity ranges from 0 (the two states have completely non-overlapping support) to 1 (they are identical). It gives us a concise, interpretable answer to the query related to similarity of population profiles.

**Lemma** **1.** *The population state matrix* ρ *is a valid density matrix. That is, it is Hermitian, positive semi-definite, and has a trace of 1.*

**Proof.**  With reference to Equation (2)

1. Hermitian: The outer product $\left|\Psi_{k}\rangle \langle \Psi_{k}\right|$ is Hermitian, as $\left(|\Psi_{k}\rangle \langle \Psi_{k}|{)}^{†}=\langle \Psi_{k}{|}^{†}|\Psi_{k}\rangle^{†}=|\Psi_{k}\rangle \langle \Psi_{k}\right|$. Therefore, ρ, being a sum of Hermitian matrices, is also Hermitian.
2. Positive Semidefinite: For any arbitrary state vector |v⟩ , the expectation value is $\langle v|\rho |v\rangle =\frac{1}{N}\sum_{k=1}^{N}\langle v|\Psi_{k}\rangle \langle \Psi_{k}|v\rangle =\frac{1}{N}\sum_{k=1}^{N}|\langle \Psi_{k}|v\rangle {|}^{2}\geq 0$. Thus, ρ is positive semi-definite.
3. Trace is 1: The trace of the outer product $\left|\Psi_{k}\rangle \langle \Psi_{k}\right|$ is $\mathrm{Tr}(|\Psi_{k}\rangle \langle \Psi_{k}|)=\langle \Psi_{k}|\Psi_{k}\rangle =1$, since the state vectors are normalized. Therefore, $\mathrm{Tr}(\rho )=\mathrm{Tr}(\frac{1}{N}\sum_{k=1}^{N}|\Psi_{k}\rangle \langle \Psi_{k}|)=\frac{1}{N}\sum_{k=1}^{N}\mathrm{Tr}(|\Psi_{k}\rangle \langle \Psi_{k}|)=\frac{1}{N}\sum_{k=1}^{N}1=1.$

As per the von Neumann entropy [13,14] Appendix A:

(8) $$S(\rho_{X})=-\sum_{i}\lambda_{i}{log}_{2}\lambda_{i}=-\sum_{i}p(x_{i}){log}_{2}p(x_{i})=H(X)$$

So, for the kind of data we are working with—categorical, classical—the von Neumann entropy is exactly the Shannon entropy, no more and no less. □

**Lemma** **2.** *Let* $P=\{p_{1},p_{2},\ldots ,p_{n}\}$ *and* $Q=\{q_{1},q_{2},\ldots ,q_{n}\}$ *be two discrete probability distributions representing cardiovascular risk profiles of two populations. If the quantum Jensen–Shannon divergence is defined as* $D_{QJSD}(P∥Q)=\frac{1}{2}\left(S\left(P∥M\right)+S\left(Q∥M\right)\right),$ *where* $M=\frac{1}{2}(P+Q)$ *and* S(⋅∥⋅) *is the quantum relative entropy, then* $0\leq D_{QJSD}(P∥Q)\leq log2$.

**Theorem** **1.** *Known as subadditivity of entropy, states that the uncertainty of a joint population state is never larger than the combined uncertainties of its individual parts.*

**Proof for Theorem** **1.** (For any bipartite population state $\rho_{AB}$ representing two risk factors A and B, $S(\rho_{AB})\;\leq \;S(\rho_{A})+S(\rho_{B}))$, has been explained briefly in the Supplementary File. Theorem 1 shows that the combined uncertainty of two risk factors like diabetes and hypertension can never exceed the sum of their individual uncertainties. In epidemiological terms, this means that correlation between risk factors reduces overall uncertainty, since their joint distribution contains shared information. Studying them together therefore yields strictly more insight than analyzing each separately, which explains why systemic approaches are more effective at capturing comorbidities than factor-by-factor methods. □

The next result, Theorem 2, formalizes joint concavity of the square root fidelity under convex mixing of density operators. This ensures that fidelity-based similarity measures remain stable when populations are formed as weighted mixtures of subgroups.

**Theorem** **2.** *Let* $\rho_{1},\rho_{2},\sigma_{1},\sigma_{2}$ *be density matrices on a finite-dimensional Hilbert space and let* λ∈[0,1]*. Define the convex mixtures* $\rho =\lambda \rho_{1}+(1-\lambda )\rho_{2},\;\;\sigma =\lambda \sigma_{1}+(1-\lambda )\sigma_{2}.$ *Then the square-root fidelity is jointly concave:* $\sqrt{F(\rho ,\sigma )}\;\geq \;\lambda \sqrt{F(\rho_{1},\sigma_{1})}+(1-\lambda )\sqrt{F(\rho_{2},\sigma_{2})}.$ *Equivalently, the map* $(\rho ,\sigma )\mapsto \sqrt{F(\rho ,\sigma )}$ *is concave on the convex set of pairs of density operators.*

The Proof for the same has been explained in the Supplementary File for reference. Theorem 2 guarantees that the fidelity remains stable under convex mixing of populations. In other words, pooling cannot make two populations look artificially different. This is important because real cohorts are almost always heterogeneous, and we need to know that our similarity scores like the F≈0.76 we report later are not being dragged down by the act of aggregation itself.

We now turn to a property that, while perhaps intuitive, needs to be stated precisely. Theorem A1 confirms that the Systemic Mutual Information between any two risk factors can never be negative. In plain terms: correlations can only carry information, they can never “subtract” it. A zero value simply means the two factors are independent.

**Theorem** **3.** *Let* P *and* Q *be population state matrices (density operators) modeling cardiovascular disease (CVD) profiles for Indian and global populations, respectively. Define the quantum Jensen–Shannon divergence (QJSD) by* $D_{QJSD}(P∥Q)\;=\;S\;\left(P+Q/2\right)\;-\;1/2S(P)\;-\;1/2S(Q),$ *where* $S(\cdot )=-Tr(\cdot {log}_{2}\cdot )$ *is the von Neumann entropy (log base* 2*). Then*

1. $D_{QJSD}(P∥Q)\geq 0$.
2. $D_{QJSD}(P∥Q)\leq 1$.
3. $D_{QJSD}(P∥Q)=0$ *if and only if* P=Q*, and the maximum* $D_{QJSD}(P∥Q)=1$ *is attained when* P *and* Q *are pure states with orthogonal supports.*

**Proof.**  For non-negativity, let M=1/2(P+Q). Since von Neumann entropy is concave,

(9) S(M) ≥ 1/2S(P)+1/2S(Q),

which yields $D_{QJSD}(P∥Q)\geq 0$.

For the upper bound, $D_{QJSD}$ equals the Holevo information of the ensemble {(1/2,P),(1/2,Q)} [15,16]:

(10) $$D_{QJSD}(P∥Q)=S(M)-1/2S(P)-1/2S(Q)\ldots$$

The Holevo quantity for two equiprobable states is bounded by the Shannon entropy of {1/2,1/2}, which is 1 bit. Hence $D_{QJSD}(P∥Q)\leq 1$. The maximum is realized when P and Q are orthogonal pure states: in that case S(P)=S(Q)=0 and S(M)=1.

For equality conditions, $D_{QJSD}(P∥Q)=0$ holds precisely when P=Q (since entropy concavity is strict unless the states coincide). The maximum value 1  occurs for orthogonal pure states, as described above. □

What these properties buy us in practice is a scale-defined divergence measure. If the QJSD between two synthetic populations is close to zero, this would suggest that a health intervention designed for one synthetic population could plausibly work for the other without much adaptation. A value near 1 bit, in contrast, would be a warning sign: the two populations differ so much that strategies borrowed from one to the other are unlikely to be useful. The bounded nature of the measure also allows easy comparison of divergence scores between different pairs of populations or different subsets of risk factors.

An important sanity check for any correlation measure is whether it can be inflated by the way you preprocess the data, and shows that no amount of local post-processing—binning, merging categories, or any other kind of coarse-graining can ever *increase* the SMI. Correlations can only decrease or stay the same.

More precisely, let $\rho_{AB}$ be the joint density matrix for two risk factors A and B in the synthetic population, with marginals $\rho_{A}={Tr}_{B}(\rho_{AB})$ and $\rho_{B}={Tr}_{A}(\rho_{AB})$. Their mutual information is $I(A:B{)}_{\rho }=S(\rho_{A})+S(\rho_{B})-S(\rho_{AB}),$ where S(ρ)=−Tr(ρlogρ) is the von Neumann entropy.

If $\Phi_{A}$ and $\Phi_{B}$ are any CPTP maps (for instance, stochastic processing, measurement, or category aggregation) applied locally to A and B, producing transformed variables A’ and B’ with joint state $\rho_{A’B’}=(\Phi_{A}⨂\Phi_{B})(\rho_{AB}).$ Then, $I(A:B{)}_{\rho }\;\;\geq \;\;I(A^{’}:B^{’}{)}_{\rho_{A’B’}}.$

In practical terms, this means that you cannot create correlations that did not already exist by playing with the data. For example, if we decide to combine “light smokers” and “heavy smokers” into one group, “smoker,” then the SMI between smoking and hypertension can only decrease or stay the same; it can never increase. This is an important safeguard: it tells us that any comorbidity structure we find is a real feature of the data, not an artifact of how we decided to bin or aggregate our variables. Any decrease in SMI after post-processing is indicative of a true resolution loss and not an analysis artifact. We show that the mutual information between any two factors can be at most twice the entropy of the smaller of the two.

Let $\rho_{AB}$ denote the joint density matrix of two risk factors A and B, with reduced states $\rho_{A}={Tr}_{B}(\rho_{AB})$ and $\rho_{B}={Tr}_{A}(\rho_{AB})$. The Systemic Mutual Information is [27,28] presented as per Equation (6).

Then the following bound holds:

(11) $$I(A:B{)}_{\rho }\;\leq \;2\;min\;\{S(\rho_{A}),\;S(\rho_{B})\}\ldots$$

The intuition here is straightforward. The “smaller” variable is a bottleneck. If one risk factor, say smoking status, has very little entropy (because almost everyone in the population is a non-smoker), then there is only so much information it can possibly share with a second, richer variable like blood pressure. In other words, a low-diversity variable simply cannot be physically involved in a high-information correlation. The bound is two times its entropy. In practice the actual SMI will be far below that.

In mathematical language, fidelity is monotone under any CPTP map, including the partial trace.

Fidelity never decreases under quantum operations: if we apply any completely positive trace-preserving (CPTP) map to a pair of states, their similarity can only go up or remain unchanged. As a special case, discarding part of the system via partial trace can only make two reduced states look more similar, not less.

Let ρ and σ be density matrices on a Hilbert space H. Recall that the Uhlmann fidelity with respect to Equation (7).

Now suppose Φ:B(H)→B(H’) is a CPTP map—this could be anything from a noisy channel to a coarse-graining of categories. Then fidelity obeys the following monotonicity property [30,31]:

(12) $$F\left(\Phi (\rho ),\Phi (\sigma )\right)\;\;\geq \;\;F(\rho ,\sigma )$$

This property has a very concrete implication for anyone comparing populations. When you look at the full six-factor profile, the fidelity between Indian and global synthetic population might be moderate. But if you then narrow your focus to, say, just diabetes and hypertension, the fidelity for that reduced pair is guaranteed to be at least as high. In other words, marginal or subset analyses tend to smooth over differences. This is precisely why it matters to study the full multi-factor state: it is the only level at which the true extent of population divergence is visible [4,5].

## 3. Results

With the theoretical machinery now in place, we turn to the numbers. This section walks through the results of applying our framework to the Indian and global synthetic population, covering single-factor entropy, pairwise mutual information, and cross-population fidelity.

### 3.1. Descriptive Statistics and Initial Data Analysis

Before getting into the information-theoretic measures, we find it instructive to look at the raw prevalence figures. Table 2 presents baseline rates for three important binary risk factors. The Indian synthetic population had a higher prevalence of diabetes (30%) than the global synthetic population (15%) by a factor of two, whereas the global synthetic population was ahead on smoking (25% vs. 20%). These differences already point to the different disease architectures we set out to characterize, and the following entropy-based analysis will make them precise.

### 3.2. Analysis of Systemic Uncertainty via Entropy

**Figure 3:**
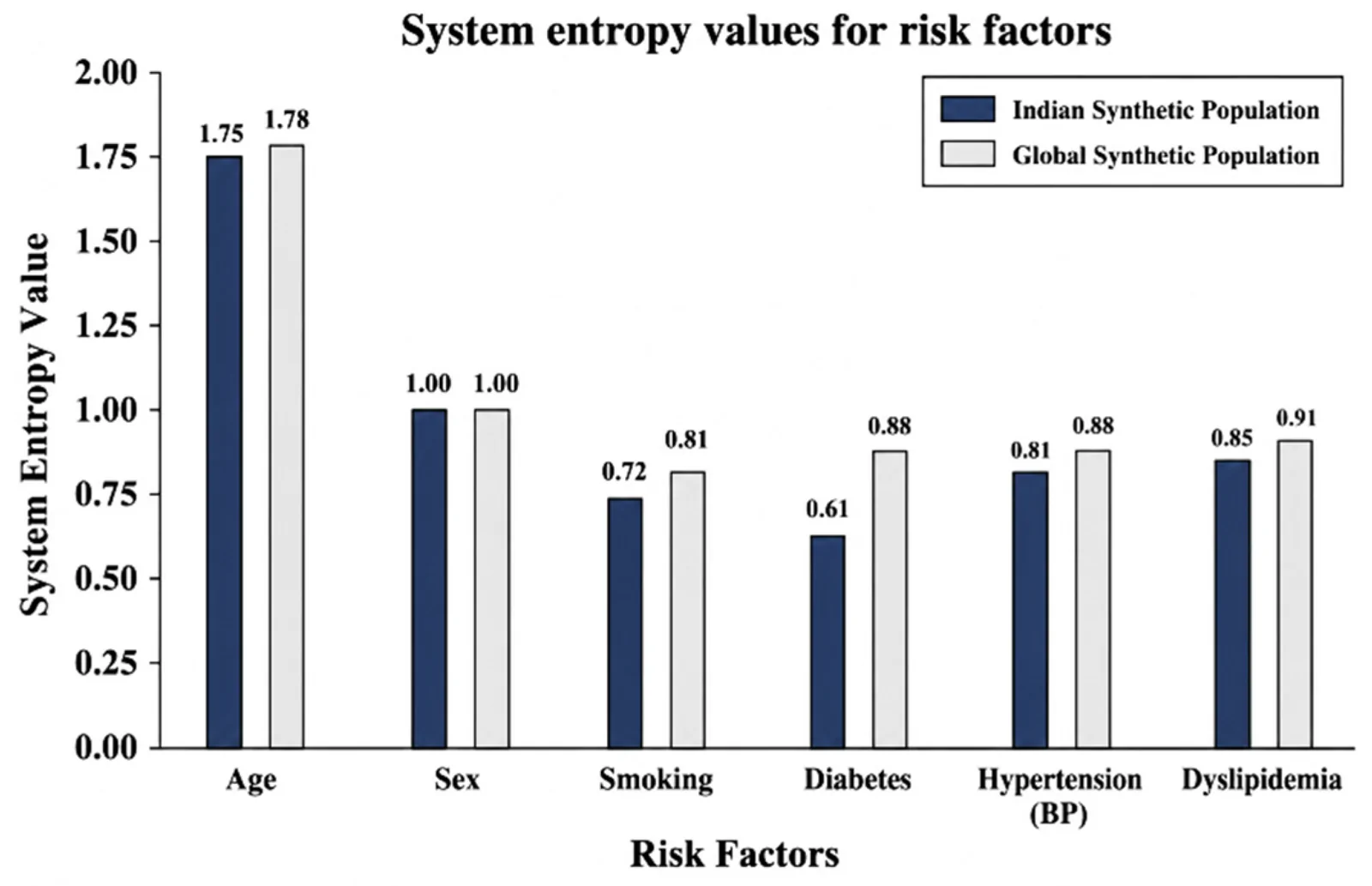
System Entropy values for risk factors.

The System Entropy $S(\rho_{X})$ tells us, for each risk factor, how spread out the population is across the possible categories. A high value—close to the theoretical maximum of 1 bit for a binary variable—means the population is nearly evenly split, so predicting any individual’s status is hard. A low value means one category dominates. Table 3 and Figure 3 lay out the results, and one contrast jumps out immediately.

The key finding here is diabetes. The Indian synthetic population has a System Entropy of 0.88 bits, which is much higher than the global synthetic population’s 0.61 bits. What this practically means is that the Indian population is much closer to an even split between diabetic and non-diabetic individuals (the distribution is more balanced, so each individual’s status is harder to guess). This is a testament to the high and pervasive occurrence of diabetes in India: the risk is not limited to a small cluster of people but is spread throughout the population, which makes it impossible to make general assumptions about any particular synthetic individuals. Conversely, smoking entropy is higher in the global synthetic population (0.81 bits vs. 0.72 bits), indicating a more balanced distribution of smokers and non-smokers and implying that smoking is a larger population-wide issue in the global context.

Figure 3 presents the bar chart of System Entropy values for each risk factor in the Indian and Global synthetic population. Higher values indicate more uncertainty or diversity across the population for that factor.

This shows the variation in entropy values of major cardiovascular risk factors in Indian and global population. The higher values seen in the global synthetic population indicate increased heterogeneity and variability in lifestyle and metabolic traits. Symmetric Mutual Information (SMI) for Joint Entropy and Comorbidity Mapping is shown in Figure 3. Side-by-side visualization of entropy values is presented in Table 3. The risk factor rankings are broadly similar in both synthetic populations but the global synthetic population reflects more diverse lifestyle and metabolic profiles across populations.

Table 4 shows the joint entropy of some pairs of risk factors. As predicted by the Theorem A2 (subadditivity), each joint entropy is greater than or equal to the entropy of either factor. The pair diabetes–hypertension has the highest joint entropy in both synthetic populations, confirming that the population-level variability is the greatest in the co-occurrence of these two conditions.

**Figure 4:**
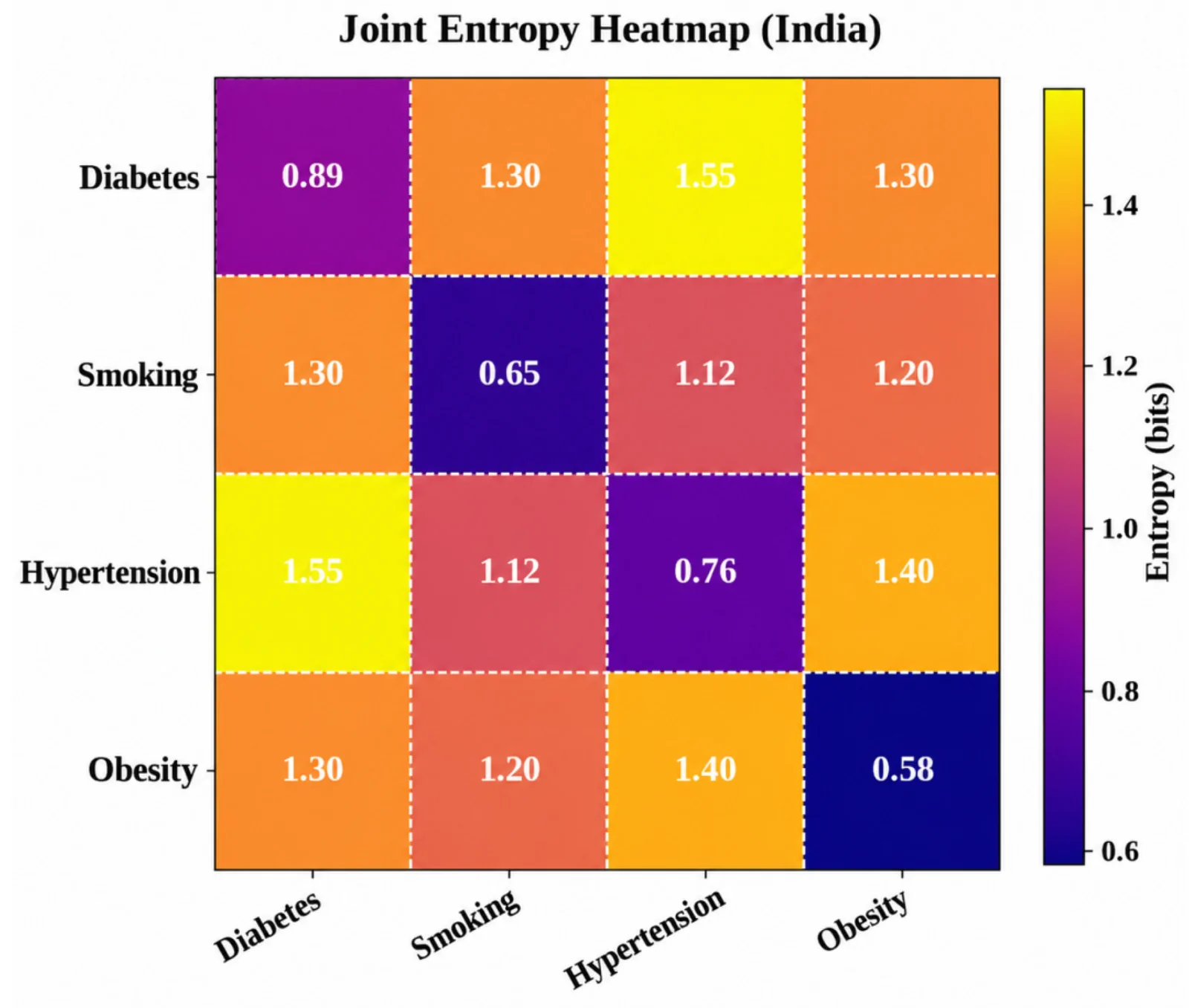
Heatmap of joint entropy values (India).

The pattern can be easily read at a glance in the heatmap shown in Figure 4. Darker tiles indicate higher joint uncertainty, with the diabetes–hypertension cell clearly in the lead, and the smoking–obesity cell having comparatively modest values.

This picture reinforces the picture of a metabolic risk cluster which is far more variable, and therefore far more clinically unpredictable, than the lifestyle-oriented pairings.

**Figure 5:**
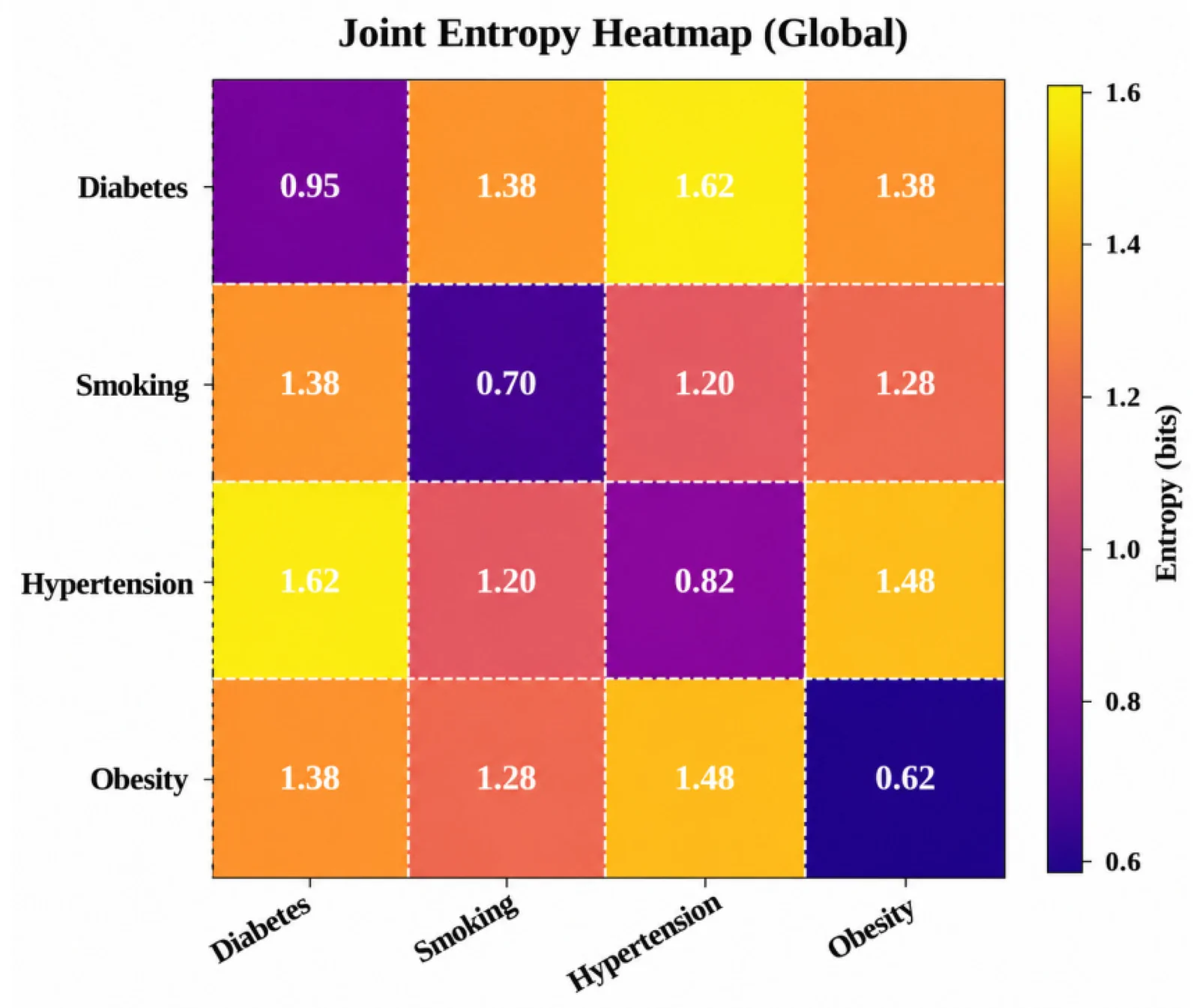
Heatmap of joint entropy values (global).

The same heatmap for the global synthetic population is shown in Figure 5. The general pattern is similar to the Indian one in broad strokes, with diabetes hypertension again at the top, but the overall values tend to be slightly higher across the board, consistent with the greater heterogeneity we saw in the single-factor entropies.

The persistent dominance of the diabetes–hypertension pair in both populations highlights its central role in cardiovascular risk, irrespective of geographic location.

The diabetes–hypertension SMI is the most informative single number in this sub-section. In the Indian synthetic population, it is 0.46 bits. This is more than twice the global value of 0.22 bits. More concretely, if you know whether an Indian synthetic individual has diabetes, your uncertainty about whether they have high blood pressure drops by nearly a half a bit, on average. This level of coupling is a quantitative signature of metabolic syndrome, in which insulin resistance, hyperglycemia and high blood pressure reinforce each other in a tight feedback loop. The world synthetic population is a different story. Here the strongest coupling is seen between smoking and hypertension (0.36 bits as opposed to 0.21 for India). This indicates that in predominantly western populations, lifestyle factors have a more independent and significant impact on blood pressure.

One natural concern with any discretized analysis is whether the results could be artifacts of the binning scheme that we have chosen. Corollary 1 removes that concern. It shows that if we apply the same coarse-graining—merging bins, reducing resolution—to both synthetic populations, the observed India-vs-Global SMI gap can only shrink, never grow. The large gaps we report above are, if anything, conservative estimates of the true structural disparity.

**Corollary** **1.** *Let* A,B *be risk factors (e.g., Diabetes and BP), and suppose the same bin-merging/coarse-graining channel* $\Phi_{A}⨂\Phi_{B}$ *is applied to both synthetic populations. Then* $I(A:B{)}_{\mathrm{India}}-I(A:B{)}_{\mathrm{Global}}\;\geq \;I(A’:B’{)}_{\mathrm{India}}-I(A’:B’{)}_{\mathrm{Global}}.$ *Therefore, observed India* > *Global SMI gaps for a pair* (A,B) *cannot be artifacts created by coarser discretization; if anything, coarse-graining can only shrink these gaps. Proof of the above statement is presented in the Supplementary File for reference.*

In other words, the gap between the two synthetic populations’ mutual information can only shrink (or stay the same) when we coarsen the data. If India shows a higher SMI than the global synthetic population in the fine-grained data, that difference is genuine—using broader bins cannot have created it.

Fidelity-Based Population Similarity and Divergence:

Table 5 puts a number on the overall similarity between the two synthetic populations. A fidelity of 0.76 for the overall population means that there is substantial overlap in the risk-factor distributions.

The urban sub-populations are more similar (0.78), while rural sub-populations diverge more (0.72). This urban–rural split is noteworthy: it suggests that urbanization tends to push risk profiles toward a common global pattern, while rural populations retain more of their region-specific disease architecture.

**Figure 6:**
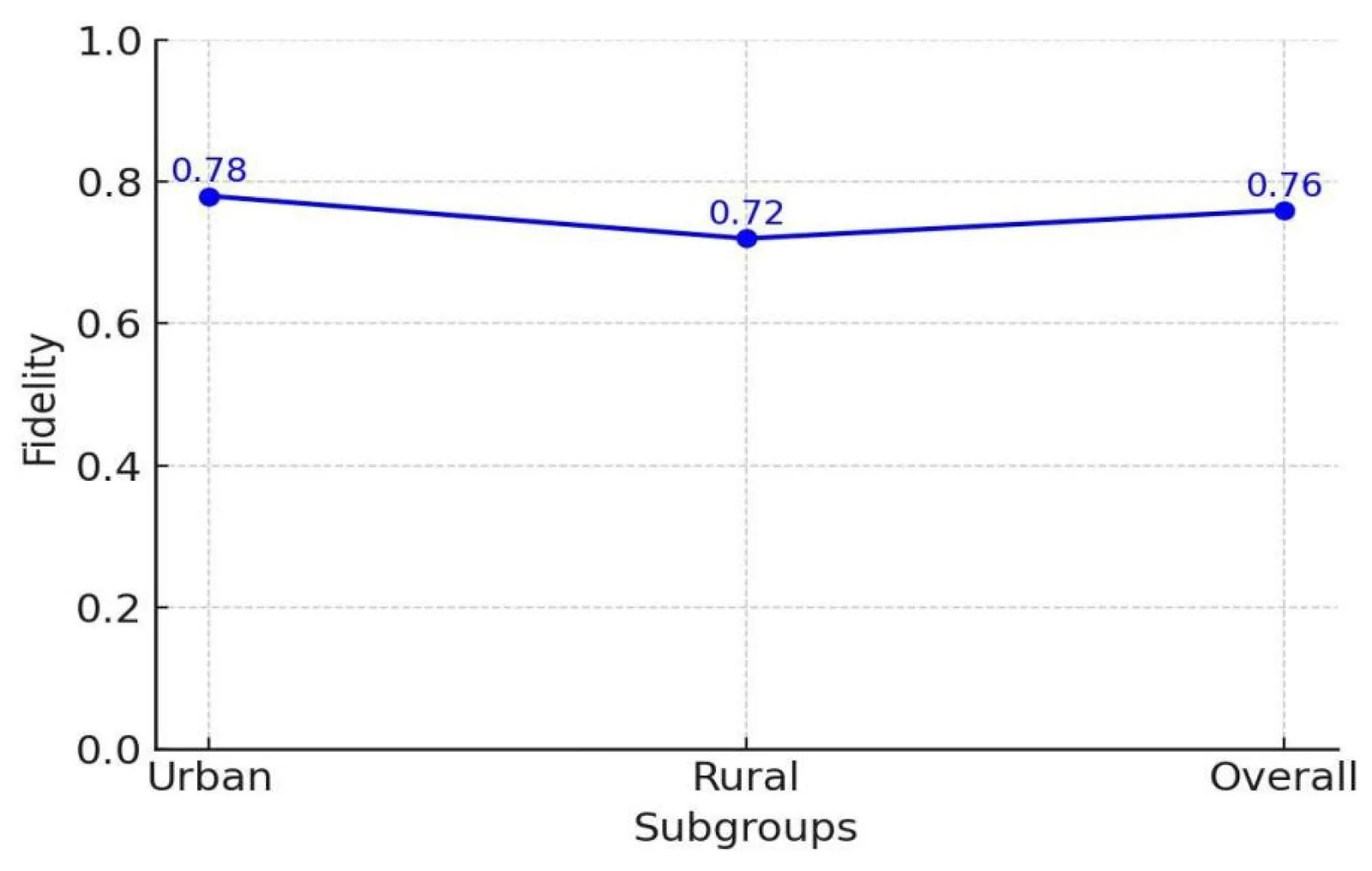
Fidelity comparisons across subgroups.

This pattern is obvious at a glance in Figure 6. The height of the urban bar is visibly higher, supporting the idea that global standard health interventions may be more easily transferred to urban Indian settings. On the other hand, rural populations may require interventions tailored to their own metabolic risk architecture. This is consistent with the concavity property of fidelity proved in Theorem A3: the total score of the mixture synthetic population cannot be less than the weighted average of the subgroup scores, and the bar chart confirms that this theoretical guarantee is observed in the data. 

A consequence of the monotonicity property that is interesting is that the whole-system fidelity is a lower bound for every marginal comparison. In Corollary 2 we can state it as follows. Since the total fidelity is close to 0.76, no individual risk factor, whether it be age, sex, smoking, diabetes, blood pressure or cholesterol, can have a marginal fidelity less than 0.76. Every subset of factors is equally considered.

**Corollary** **2.** *Given the reported whole-system fidelity* $F(\rho_{\mathrm{India}},\rho_{\mathrm{Global}})\approx 0.76$*, every single-factor marginal* X *(Age, Sex, Smoking, Diabetes, BP, Chol) satisfies* $F\;\left((\rho_{\mathrm{India}}{)}_{X},\;(\rho_{\mathrm{Global}}{)}_{X}\right)\;\geq \;0.76.$ *More generally, any subset of factors has fidelity at least* 0.76*. Proof of Corollary 2 is explained in the Supplementary File.*

Any policy that considers the Indian risk architecture and the global risk architecture as equivalent is actually ignoring a quarter of the systemic changes our framework detects. To make the divergence even more tangible, Corollary 3 translates the fidelity score into bounds on the trace distance, a norm-based measure that has a role similar to total variation in classical statistics. This resulting interval tells us exactly how far apart the two population states are in terms of trace norm, and the fact that the lower bound is strictly positive confirms that the two synthetic populations are indeed statistically distinguishable.

**Corollary** **3.** *Trace-distance bounds from fidelity: let* $D(\rho ,\sigma ):=1/2∥\rho -\sigma {∥}_{1}$ *be the trace distance. The Fuchs–van de Graaf inequalities yield* $1-\sqrt{F(\rho ,\sigma )}\;\leq \;D(\rho ,\sigma )\;\leq \;\sqrt{1-F(\rho ,\sigma )}.$ *With* $F(\rho_{\mathrm{India}},\rho_{\mathrm{Global}})\approx 0.76$*, we obtain* $0.129\;≲\;D(\rho_{\mathrm{India}},\rho_{\mathrm{Global}})\;≲\;0.490.$ *Thus the synthetic populations differ by a nontrivial total-variation–like distance, with a* 24% *systemic discrepancy (Proof is explained in the Supplementary File).*

We apply these two scalar inequalities directly to the reported whole-system fidelity $F(\rho_{\mathrm{India}},\rho_{\mathrm{Global}})\approx 0.76$. Since both bounding functions $F\mapsto 1-\sqrt{F}$ and $F\mapsto \sqrt{1-F}$ are continuous and monotone on [0,1], substituting F≈0.76 gives numerical bounds on D:

Lower bound:

$$1-\sqrt{F}\;\approx \;1-\sqrt{0.76}\;=\;1-0.8717797887\;\;=\;0.1282202113\;\approx \;0.128.$$

Upper bound:

$$\sqrt{\;1-F\;}\;\approx \;\sqrt{\;1-0.76\;}\;=\;\sqrt{0.24}\;=\;0.4898979485\;\;\;\approx \;0.490.$$

Rounding to three significant digits yields

$$0.129\;≲\;D(\rho_{\mathrm{India}},\rho_{\mathrm{Global}})\;≲\;0.490,$$

as claimed. The nonzero lower bound shows a strictly positive trace distance (hence statistical distinguishability). The appearance of 1−F=0.24 in the upper bound reflects that for pure states, the FvG upper bound is tight, i.e., $D=\sqrt{1-F}$.

## 4. Discussion

*Interpretation of Key Findings and Numerical Implications*

The numerical results signify the modeled CVD risk factor architecture with reference to populations. The diabetes–hypertension SMI of 0.46 bits in the Indian synthetic population indicates stronger statistical dependence in the constructed model, consistent with integrated cardiometabolic assessment but not evidence of causality or screening policy without clinical data analysis. High diabetes entropy (0.88 bits) reflects modeled uncertainty driven by prevalence inputs.

*Clinical and Public Health Policy Implications*

The policy implications of the two comorbidity architectures are different, and they differ between the two populations: For India, the strong metabolic coupling has documented and strongly argued for integrated screening and management programs that address diabetes, hypertension and dyslipidemia together rather than in siloed campaigns. For example, a diabetes screening campaign should automatically include blood pressure checks if it is to get a complete picture of the risks.

For global health agencies such as the WHO, our data show that drivers of CVD are not all the same. The higher smoking–hypertension SMI in the global synthetic population suggests that anti-smoking campaigns in predominantly Western populations would have a more direct, measurable impact on blood-pressure-related cardiovascular events than similar campaigns in South Asia, where the metabolic pathways dominate.

## 5. Methodological Strengths and Novelty

From a methodological perspective, the main contribution of this work is to quantify relationships that are often discussed only qualitatively, in precise, interpretable numbers. The SMI metric is able to capture the full statistical dependence structure between any pair of variables, much more than a simple correlation coefficient can express. The fidelity score takes a complicated, high-dimensional comparison and collapses it into a single number that can be readily understood by policy-makers and clinicians. And since each measure is based on the well-developed theory of quantum information, we inherit a suite of rigorous properties (positivity, data-processing inequalities, monotonicity) that give us confidence in the robustness of the results.

## 6. Limitations and Future Work

That being said, there are clear limitations to the current analysis, and we want to be upfront about them. The most obvious one is that our framework views the population as a snapshot: we capture the risk-factor distribution at a single point in time, and say nothing about the way it changes. A natural extension is to construct time-dependent state matrices, ρ(t), and track fidelity and SMI over successive years. This would allow us to quantify if a public health intervention actually decouples closely linked risk factors or reduces total systemic entropy, effectively providing a quantitative report card on policy effectiveness.

## 7. Conclusions

This proposed work explained a quantum inspired framework for comparing CVD risk profiles. The behavior of cardiovascular diseases with respect to populations has projected by considering all the correlation factors. Two fundamentally dissimilar types of disease architectures in Indian synthetic population and the global one have been analyzed precisely. Illustrative results suggested stronger diabetes–hypertension dependence in the Indian scenario and stronger smoking–hypertension dependence in the global comparator, with whole system fidelity F≈0.76. These are proof-of-concept point estimates. Clinical measures. In terms of the novelty of this research, it was primarily considered a demonstration of a new approach’s applicability. Indeed, we are able to utilize techniques developed within quantum information theory to yield some practical results regarding epidemiology. The figures provided above were calculated on the basis of modeled data, but now the task is to prove our approach’s validity through application to real-world data. In case of success, this technique will definitely take its place among others in comparative epidemiology practice.

## Figures and Tables

**Table 1:** Variable definitions and discretization strategy.

Variable	Description	Categories and Encoding
Age	Chronological age.	4 Bins: 20–39 (vector 1), 40–59 (vector 2), 60–79 (vector 3), ≥80 (vector 4).
Sex	Biological sex.	2 Bins: Female (vector 1), Male (vector 2).
Smoking	Tobacco smoking status.	2 Bins: Non-Smoker (vector 1), Current Smoker (vector 2).
Obesity	BMI.	2 Bins: Non-Obesity (vector 1), Obesity (Vector 2)
Diabetes	Diabetes Mellitus status.	2 Bins: Normoglycemic (vector 1), Diabetic (vector 2).
Hypertension (BP)	Blood pressure status.	4 Bins: Normal (vector 1), Elevated (vector 2), Stage 1 HTN (vector 3), Stage 2 HTN (vector 4).
Dyslipidemia (Cholesterol)	Cholesterol status.	4 Bins: Desirable (vector 1), Borderline High (vector 2), High (vector 3), Very High (vector 4).

**Table 2:** Baseline prevalence of key binary factors in modeled synthetic populations.

Risk Factor	Prevalence (%) (Indian Synthetic Population)	Prevalence (%) (Global Synthetic Population)
Diabetes	30.0%	15.0%
Smoking	20.0%	25.0%
Hypertension (Stage 1+)	30.0%	25.0%

**Table 3:** System Entropy analysis for all risk factors (in bits).

Risk Factor	System Entropy—Indian	System Entropy—Global
Age	1.75	1.78
Sex	1.00	1.00
Smoking	0.72	0.81
Diabetes	0.61	0.88
Hypertension (BP)	0.81	0.88
Dyslipidemia	0.85	0.91

**Table 4:** Joint entropy of selected risk factor pairs.

Risk Factor Pair	India (Bits)	Global (Bits)
(Diabetes, Hypertension)	1.30	1.50
(Smoking, Obesity)	1.12	1.20
(Diabetes, Obesity)	1.30	1.38

**Table 5:** Fidelity values between Indian and global synthetic population.

Subgroup	Fidelity
Urban (India vs. Global)	0.78
Rural (India vs. Global)	0.72
Overall Population	0.76

## Data Availability

The public synthetic population data presented in this study are available with the CVD-synthetic population-data repository at https://github.com/let-the-dreamers-rise/cvd-cohort-data (accessed on 1 November 2025).

## Supplementary Materials

The following supporting information can be downloaded at: https://www.mdpi.com/article/10.3390/diagnostics16172878/s1, File S1: Cardiovascular Disease Profiles.

## Appendix A

**Lemma** **A1.** *For a risk factor* X *with a probability distribution* $p(x_{i})$*, its classical Shannon entropy* H(X) *is equal to the von Neumann entropy* $S(\rho_{X})$ *of its corresponding reduced state matrix.*

**Proof.** The reduced state matrix $\rho_{X}$ is obtained by tracing out all other subsystems. Because our initial state vectors are formed from orthogonal bases, the resulting matrix $\rho_{X}$ is a diagonal matrix where the diagonal elements are the marginal probabilities of the states of X. Thus, $\rho_{X}=\mathrm{diag}(p(x_{1}),p(x_{2}),\ldots ,p(x_{d_{X}}))$. The eigenvalues of a diagonal matrix are its diagonal elements. □

## Appendix B

**Theorem** **A1.** *The Systemic Mutual Information* I(A:B) *between two risk factors* A *and* B *is always non-negative:* $I(A:B)\;=\;S(\rho_{A})+S(\rho_{B})-S(\rho_{AB})\;\geq \;0.$ *Proof for Theorem A1 has been explained briefly in the Supplementary File for reference.*

Theorem A1 shows that Systemic Mutual Information never takes negative values. If I(A:B)=0, the two risk factors are statistically independent, meaning that observing one provides no knowledge of the other. If I(A:B)>0, the factors are correlated, and learning about one reduces uncertainty about the other. Larger values of I(A:B) signal stronger comorbidities or systemic couplings, such as the well-documented clustering of diabetes and hypertension in synthetic individuals’ populations.

Theorem A2 restates and strengthens this point from a slightly different angle, making the connection to relative entropy explicit. The joint uncertainty of any two factors is at most the sum of their individual uncertainties, and the gap between the two is precisely the mutual information.

**Theorem** **A2.** *For any bipartite state* $\rho_{AB}$ *with marginals* $\rho_{A}={Tr}_{B}(\rho_{AB})$ *and* $\rho_{B}={Tr}_{A}(\rho_{AB})$*, the Systemic Mutual Information is always non-negative:* $I(A\;:\;B)=S(\rho_{A})+S(\rho_{B})-S(\rho_{AB})\;\geq \;0,$ *with equality iff* $\rho_{AB}=\rho_{A}⨂\rho_{B}$*. Theorem A2 proof has summarized in Supplementary File for reference.*

The upshot of Theorem A2 is that SMI gives us a clean, unambiguous readout of how much two risk factors depend on each other. A value of zero pins down exact independence—the joint distribution factors neatly into the product of its margins. Any positive value quantifies a departure from that baseline, with bigger numbers pointing to tighter comorbidity. Think of it as a ruler for measuring how much knowing a synthetic individuals’ diabetic status would help you predict their blood pressure category, or vice versa. It is exactly this kind of hidden dependency structure that traditional regression models tend to undercount.

Next, Theorem A3 pins down the basic properties one would want from any sensible similarity measure, applied here to fidelity. It confirms that the score is always between 0 and 1, that you get a perfect 1 only when the two population states are identical, and that it drops to 0 when the two profiles have completely non-overlapping support.

**Theorem** **A3.** *For any two density operators* ρ *and* σ *on the same finite-dimensional Hilbert space, the Uhlmann–Jozsa fidelity* $F(\rho ,\sigma )\;=\;(Tr\sqrt{\sqrt{\rho }\;\sigma \;\sqrt{\rho }}{)}^{2}$ *satisfies* 0≤F(ρ,σ)≤1. *Moreover,* F(ρ,σ)=1 *if and only if* ρ=σ*, and* F(ρ,σ)=0 *when the supports of* ρ *and* σ *are orthogonal. Proof for Theorem A3 has explained in Supplementary File for reference.*

In practice, fidelity provides a single number that characterizes the degree of overlap between two population profiles. A score close to 1 indicates that their joint risk-factor distributions are almost indistinguishable; a score close to 0 indicates that the two profiles share few commonalities. For our purposes of comparing Indian and global CVD architectures, this is invaluable as it reduces a high dimensional comparison to a single interpretable quantity.

Besides fidelity, we also introduce the quantum Jensen–Shannon divergence (QJSD) which measures the difference between two population profiles, i.e., the opposite of fidelity. Theorem 3 shows that this divergence behaves well: it is non-negative, never exceeds 1 bit, and is zero only when the two profiles are the same.
