# Supplementary material for: Synthetic Comparative Analysis of Cardiovascular Disease Profiles Using Quantum Information Measures in Indian and Global Populations
Source: Diagnostics (Basel). 2026 Sep 7;16(17):2878. doi: 10.3390/diagnostics16172878 (PMC13564558; doi:10.3390/diagnostics16172878)
Supplement: Supplementary file 1 [file diagnostics-16-02878-s001.zip › Supplementary File S1.pdf]

Comparative Analysis of Cardiovascular Disease Profiles Using Quantum Information Measures in Indian and Global Populations

Supplementary Information

Theorem S1. *For any bipartite population state* ρ_{AB} *representing two risk factors* A *and* B, $S(\rho_{AB}) \le S(\rho_A) + S(\rho_B)$.

Proof. Recall the quantum (von Neumann) relative entropy between two density matrices ρ and σ, $D(\rho\|\sigma) = \mathrm{Tr}[\rho(\log\rho - \log\sigma)]$,..(S1)

which satisfies Klein's inequality (positivity): $D(\rho\|\sigma) \ge 0$,

with equality iff $\rho = \sigma$ on the support of ρ.

Apply this fact to the pair $(\rho_{AB}, \rho_A \otimes \rho_B)$, where $\rho_A = \mathrm{Tr}_B\rho_{AB}$ and $\rho_B = \mathrm{Tr}_A\rho_{AB}$. By definition $D(\rho_{AB}\|\rho_A \otimes \rho_B) = \mathrm{Tr}[\rho_{AB}\log\rho_{AB}] - \mathrm{Tr}[\rho_{AB}\log(\rho_A \otimes \rho_B)]$ ….........................(S2)

Using the identity $\log(\rho_A \otimes \rho_B) = \log\rho_A \otimes I_B + I_A \otimes \log\rho_B$ and linearity/partial-trace properties of the trace, evaluate the second term:

$\mathrm{Tr}[\rho_{AB}\log(\rho_A \otimes \rho_B)] = \mathrm{Tr}[(\mathrm{Tr}_B\rho_{AB})\log\rho_A] + \mathrm{Tr}[(\mathrm{Tr}_A\rho_{AB})\log\rho_B]$

$= \mathrm{Tr}[\rho_A\log\rho_A] + \mathrm{Tr}[\rho_B\log\rho_B]$ …...(S3)

Hence,

$D(\rho_{AB}\|\rho_A \otimes \rho_B) = \mathrm{Tr}[\rho_{AB}\log\rho_{AB}] - \mathrm{Tr}[\rho_A\log\rho_A] - \mathrm{Tr}[\rho_B\log\rho_B]$ ….........................(S4)

Recalling the von Neumann entropy $S(\tau) = -\mathrm{Tr}(\tau\log\tau)$, we rewrite the above as

$D(\rho_{AB}\|\rho_A \otimes \rho_B) = -S(\rho_{AB}) + S(\rho_A) + S(\rho_B)$ …..(S5)

By Klein's inequality the left-hand side is nonnegative, therefore

$-S(\rho_{AB}) + S(\rho_A) + S(\rho_B) \ge 0$,..(S6)

which rearranges to the claimed subadditivity inequality

$S(\rho_{AB}) \le S(\rho_A) + S(\rho_B)$ ….. (S7)

Equality holds precisely when $\rho_{AB} = \rho_A \otimes \rho_B$ on the support of ρ_{AB}, i.e. when the joint state is a product state (no correlations).

Theorem S2. *Let* $\rho_1, \rho_2, \sigma_1, \sigma_2$ *be density matrices on a finite-dimensional Hilbert space and let* $\lambda \in [0,1]$. *Define the convex mixtures* $\rho = \lambda\rho_1 + (1-\lambda)\rho_2$, $\sigma = \lambda\sigma_1 + (1-\lambda)\sigma_2$. *Then the square-root fidelity is jointly concave:* $\sqrt{F(\rho,\sigma)} \ge \lambda\sqrt{F(\rho_1,\sigma_1)} + (1-\lambda)\sqrt{F(\rho_2,\sigma_2)}$. *Equivalently, the map* $(\rho,\sigma) \mapsto \sqrt{F(\rho,\sigma)}$ *is concave on the convex set of pairs of density operators.*

Proof. We prove the joint concavity of $\sqrt{F(\cdot,\cdot)}$ via Uhlmann's purification characterization of fidelity.

1. Uhlmann's variational formula. For any pair of density matrices α, β,

$$\sqrt{F(\alpha,\beta)} = \max_{\substack{|\Phi_\alpha\rangle, |\Phi_\beta\rangle \\ \text{purifications}}} |\langle \Phi_\alpha \mid \Phi_\beta \rangle|, \quad \text{(S8)}$$

where the maximization is over all purifications $|\Phi_\alpha\rangle$ of α and $|\Phi_\beta\rangle$ of β in a common (sufficiently large) Hilbert space. (See Uhlmann's theorem / standard QIT references.)

2. Choose near-optimal purifications for components. For $i = 1,2$, pick purifications $|\Phi_{\rho_i}\rangle$ of ρ_i and $|\Phi_{\sigma_i}\rangle$ of σ_i such that **[27]**

$$|\langle \Phi_{\rho_i} \mid \Phi_{\sigma_i} \rangle| = \sqrt{F(\rho_i, \sigma_i)} \quad \text{(up to arbitrarily small } \varepsilon > 0) \quad \text{(S9)}$$

If necessary, adjust the global phases of these purifications so that each inner product $\langle \Phi_{\rho_i} \mid \Phi_{\sigma_i} \rangle$ is a nonnegative real number (this can always be done by multiplying one purification by a phase).

3. Construct purifications for the mixtures. Introduce a two-dimensional ancilla with orthonormal basis $\{|e_1\rangle, |e_2\rangle\}$. Define the vectors **[30]**

$$|\Psi_\rho\rangle := \sqrt{\lambda}\,|\Phi_{\rho_1}\rangle \otimes |e_1\rangle + \sqrt{1-\lambda}\,|\Phi_{\rho_2}\rangle \otimes |e_2\rangle, \quad \text{(S10)}$$

$$|\Psi_\sigma\rangle := \sqrt{\lambda}\,|\Phi_{\sigma_1}\rangle \otimes |e_1\rangle + \sqrt{1-\lambda}\,|\Phi_{\sigma_2}\rangle \otimes |e_2\rangle \quad \text{(S11)}$$

Each of these is normalized because cross terms between $|e_1\rangle$ and $|e_2\rangle$ vanish. Tracing out the ancilla yields the mixtures:

$$\mathrm{Tr}_{\mathrm{anc}}\big(|\Psi_\rho\rangle\langle\Psi_\rho|\big) = \lambda\rho_1 + (1-\lambda)\rho_2 = \rho, \quad \text{(S12)}$$

and similarly $\mathrm{Tr}_{\mathrm{anc}}(|\Psi_\sigma\rangle\langle\Psi_\sigma|) = \sigma$. Thus $|\Psi_\rho\rangle$ and $|\Psi_\sigma\rangle$ are valid purifications of ρ and σ.

4. Compute the overlap of the constructed purifications. Direct inner-product computation gives**[28]**

$$\langle \Psi_\rho \mid \Psi_\sigma \rangle = \lambda\,\langle \Phi_{\rho_1} \mid \Phi_{\sigma_1} \rangle + (1-\lambda)\,\langle \Phi_{\rho_2} \mid \Phi_{\sigma_2} \rangle \quad \text{(S13)}$$

By our phase choice the right-hand terms are nonnegative real numbers, hence

$$\big|\langle \Psi_\rho \mid \Psi_\sigma \rangle\big| = \lambda\sqrt{F(\rho_1,\sigma_1)} + (1-\lambda)\sqrt{F(\rho_2,\sigma_2)} \quad \text{(S14)}$$

5. Apply Uhlmann's variational principle. Since $\sqrt{F(\rho,\sigma)}$ is the maximum absolute overlap over all purifications of ρ and σ, we have

$$\sqrt{F(\rho,\sigma)} \geq |\langle \Psi_\rho \mid \Psi_\sigma \rangle| = \lambda\sqrt{F(\rho_1,\sigma_1)} + (1-\lambda)\sqrt{F(\rho_2,\sigma_2)} \quad \text{(S15)}$$

6. Conclude. The displayed inequality is precisely the joint concavity of $\sqrt{F(\cdot,\cdot)}$, completing the proof.

To put this in epidemiological language: suppose we form a single cohort by pooling urban and rural sub-populations. Theorem S2 guarantees that the fidelity between this mixed cohort and some reference population will be at least as large as the weighted average of the sub-group fidelities. In other words, pooling cannot make two populations look artificially different. This is important because real cohorts are almost always heterogeneous, and we need to know that our similarity scores like the $F \approx 0.76$ we report later are not being dragged down by the act of aggregation itself.

Theorem S3. *The systemic mutual information $I(A{:}B)$ between two risk factors A and B is always non-negative: $I(A{:}B) = S(\rho_A) + S(\rho_B) - S(\rho_{AB}) \geq 0$.*

Proof. Let ρ_{AB} be a bipartite state with marginals $\rho_A = \mathrm{Tr}_B(\rho_{AB})$ and $\rho_B = \mathrm{Tr}_A(\rho_{AB})$. The mutual information can be expressed as a relative entropy:**[31]**

$$I(A{:}B) = S(\rho_{AB} \,\|\, \rho_A \otimes \rho_B), \quad \text{(S16)}$$

where

$$S(\rho\|\sigma) = \begin{cases} \mathrm{Tr}(\rho(\log\rho - \log\sigma)), & \text{if } \mathrm{supp}(\rho) \subseteq \mathrm{supp}(\sigma), \\ +\infty, & \text{otherwise.} \end{cases}$$

Klein's inequality ensures $S(\rho\|\sigma) \geq 0$, with equality if and only if $\rho = \sigma$. Applying this with $\rho = \rho_{AB}$ and $\sigma = \rho_A \otimes \rho_B$ gives

$$I(A{:}B) \geq 0,$$

with equality exactly when $\rho_{AB} = \rho_A \otimes \rho_B$.

The interpretation here is straightforward. When $I(A{:}B) = 0$, the two risk factors carry no information about each other they are statistically independent and knowing one tells you nothing about the other. A positive value, on the other hand, means the two factors are entangled in a statistical sense: the larger the SMI, the more tightly coupled they are. For instance, the well-known clustering of diabetes and hypertension in certain populations would show up as a notably high SMI value.

Theorem S4 restates and strengthens this point from a slightly different angle, making the connection to relative entropy explicit. The joint uncertainty of any two factors is at most the sum of their individual uncertainties, and the gap between the two is precisely the mutual information.

Theorem S4. *For any bipartite state ρ_{AB} with marginals $\rho_A = \mathrm{Tr}_B(\rho_{AB})$ and $\rho_B = \mathrm{Tr}_A(\rho_{AB})$, the systemic mutual information is always non-negative: $I(A{:}B) = S(\rho_A) + S(\rho_B) - S(\rho_{AB}) \geq 0$, with equality iff $\rho_{AB} = \rho_A \otimes \rho_B$.*

Proof. Let ρ_{AB} be a bipartite density operator with marginals $\rho_A = \mathrm{Tr}_B(\rho_{AB})$ and $\rho_B = \mathrm{Tr}_A(\rho_{AB})$. The von Neumann entropy is $S(\rho) = -\mathrm{Tr}(\rho\log\rho)$ (any fixed log base).

Step 1: Mutual information as a quantum relative entropy. Define the quantum relative entropy

$$S(\rho\|\sigma) = \begin{cases} \mathrm{Tr}(\rho(\log\rho - \log\sigma)), & \text{if } \mathrm{supp}(\rho) \subseteq \mathrm{supp}(\sigma), \\ +\infty, & \text{otherwise.} \end{cases}$$

Then

$$\begin{aligned} S(\rho_{AB}\|\rho_A \otimes \rho_B) &= \mathrm{Tr}(\rho_{AB}\log\rho_{AB}) - \mathrm{Tr}(\rho_{AB}(I_A \otimes \log\rho_B)) \\ &= -S(\rho_{AB}) - \mathrm{Tr}(\rho_A\log\rho_A) - \mathrm{Tr}(\rho_B\log\rho_B) \\ &= S(\rho_A) + S(\rho_B) - S(\rho_{AB}) \;=\; I(A{:}B). \end{aligned}$$

Step 2: Non-negativity via Klein's inequality. Klein's inequality states that for positive operators X, Y with $\mathrm{supp}(X) \subseteq \mathrm{supp}(Y)$,**[8]**

$$\mathrm{Tr}(X(\log X - \log Y)) \;\geq\; 0,$$

with equality iff $X = Y$. If $\mathrm{supp}(X) \nsubseteq \mathrm{supp}(Y)$ then $S(X\|Y) = +\infty \geq 0$ by definition.

Apply this with $X = \rho_{AB}$ and $Y = \rho_A \otimes \rho_B$:

$$I(A{:}B) = S(\rho_{AB}\|\rho_A \otimes \rho_B) \;\geq\; 0,$$

with equality iff $\rho_{AB} = \rho_A \otimes \rho_B$.

The upshot of Theorem S4 is that SMI gives us a clean, unambiguous readout of how much two risk factors depend on each other.

Theorem S5. *For any two density operators ρ and σ on the same finite-dimensional Hilbert space, the Uhlmann–Jozsa fidelity $F(\rho,\sigma) = (\mathrm{Tr}\sqrt{\sqrt{\rho}\,\sigma\,\sqrt{\rho}})^2$ satisfies $0 \leq F(\rho,\sigma) \leq 1$. Moreover, $F(\rho,\sigma) = 1$ if and only if $\rho = \sigma$, and $F(\rho,\sigma) = 0$ when the supports of ρ and σ are orthogonal.*

Proof. We present a two-part proof: (A) an expression of fidelity via purifications (Uhlmann's theorem) which immediately gives the bounds, and (B) the equivalent trace-sqrt formula.

(A) Purification representation and the bounds. Let $|\Psi_\rho\rangle$ and $|\Psi_\sigma\rangle$ be arbitrary purifications of ρ and σ in an enlarged Hilbert space $\mathcal{H} \otimes \mathcal{H}_E$, i.e.**[9]**

$\mathrm{Tr}_E(|\Psi_\rho\rangle\langle\Psi_\rho|) = \rho, \qquad \mathrm{Tr}_E(|\Psi_\sigma\rangle\langle\Psi_\sigma|) = \sigma$ ……………………………………………….(S17)

Uhlmann's theorem states that

$\sqrt{F(\rho,\sigma)} = \max_{U \text{ unitary on } \mathcal{H}_E} |\langle\Psi_\rho|(I \otimes U)|\Psi_\sigma\rangle|$ ………………………………………………….(S18)

For any fixed unitary U, the inner product $\langle\Psi_\rho|(I \otimes U)|\Psi_\sigma\rangle$ is a complex number whose absolute value is bounded by Cauchy–Schwarz:**[10]**

$|\langle\Psi_\rho|(I \otimes U)|\Psi_\sigma\rangle| \leq \| \Psi_\rho \| \, \| (I \otimes U)\Psi_\sigma \| = 1$,……………………………………………….(S19)

since purifications are unit vectors. Taking the maximum over all unitaries yields

$0 \leq \sqrt{F(\rho,\sigma)} \leq 1 \quad \Rightarrow \quad 0 \leq F(\rho,\sigma) \leq 1$ ……………………………………………….(S20)

Equality cases follow directly from the purification picture:

- If $\rho = \sigma$ then one may choose identical purifications (so $|\Psi_\rho\rangle = |\Psi_\sigma\rangle$), giving $\sqrt{F} = 1$ and hence $F = 1$.
- If the supports of ρ and σ are orthogonal then any purification of ρ is orthogonal to any purification of σ after tracing out the environment, so the overlap is zero for all unitaries and $F = 0$.

(B) Trace-sqrt representation and an alternative bound. One may equivalently write fidelity in the (often-used) operator form**[11]**

$F(\rho, \sigma) = (\mathrm{Tr}\sqrt{\sqrt{\rho}\,\sigma\,\sqrt{\rho}})^2 = \| \sqrt{\rho}\,\sqrt{\sigma}\,\|_1^2$,..(S21)

where $\|\cdot\|_1$ denotes the trace norm. The non-negativity of F is immediate from this representation since the trace of a positive operator is non-negative and the square of a real non-negative number is non-negative.

To see the upper bound from this viewpoint, note that the Hilbert–Schmidt inner product $\langle A, B\rangle_{\mathrm{HS}} = \mathrm{Tr}(A^\dagger B)$ and Cauchy–Schwarz for Hilbert–Schmidt operators give**[12]**

$\mathrm{Tr}(\sqrt{\rho}\,\sqrt{\sigma}) \leq \| \sqrt{\rho}\,\|_2 \, \| \sqrt{\sigma}\,\|_2 = \sqrt{\mathrm{Tr}\rho}\,\sqrt{\mathrm{Tr}\sigma} = 1$,...(S22)

so $\mathrm{Tr}\sqrt{\sqrt{\rho}\,\sigma\,\sqrt{\rho}} \leq 1$ and therefore $F(\rho, \sigma) \leq 1$. (The displayed inequality follows because the trace of the positive operator $\sqrt{\rho}\sqrt{\sigma}$ is bounded by its Hilbert–Schmidt norm; more generally $\| X \|_1 \leq \sqrt{\mathrm{rank}(X)}\, \| X \|_2$, and for density-operator products the combination of these norm inequalities with $\mathrm{Tr}\rho = \mathrm{Tr}\sigma = 1$ yields the stated bound.)

Equality conditions revisited.

- $F(\rho, \sigma) = 1$ iff there exists a unitary U on the environment such that $(I \otimes U)|\Psi_\sigma\rangle = |\Psi_\rho\rangle$, which (after tracing out the environment) is equivalent to $\rho = \sigma$.
- $F(\rho, \sigma) = 0$ if and only if the supports of ρ and σ are orthogonal; in that case no purification overlap is possible and the fidelity vanishes.

Corollary S1. *Let A, B be risk factors (e.g., Diabetes and BP), and suppose the same bin-merging/coarse-graining channel $\Phi_A \otimes \Phi_B$ is applied to both cohorts. Then $I(A{:}B)_{\mathrm{India}} - I(A{:}B)_{\mathrm{Global}} \geq I(A'{:}B')_{\mathrm{India}} - I(A'{:}B')_{\mathrm{Global}}$. Therefore, observed India $>$ Global SMI gaps for a pair (A, B) cannot be artifacts created by coarser discretization; if anything, coarse-graining can only shrink these gaps.*

Proof. The data-processing inequality for SMI tells us that any local coarse-graining (applying $\Phi_A \otimes \Phi_B$ to each cohort) cannot push the mutual information up. So for each cohort separately we get $I(A{:}B)_{\mathrm{India}} \geq I(A'{:}B')_{\mathrm{India}}$,.......................................(S23)

and likewise for the global cohort,$I(A{:}B)_{\mathrm{Global}} \geq I(A'{:}B')_{\mathrm{Global}}$ ....(S24)

Subtracting the second from the first gives

$I(A\!:B)_{\text{India}} - I(A\!:B)_{\text{Global}} \;\; \geq \;\; I(A'\!:B')_{\text{India}} - I(A'\!:B')_{\text{Global}}$ ....(S25)

In other words, the gap between the two cohorts' mutual information can only shrink (or stay the same) when we coarsen the data. If India shows a higher SMI than the global cohort in the fine-grained data, that difference is genuine—using broader bins cannot have created it.

Corollary S2. *Given the reported whole-system fidelity* $F(\rho_{\text{India}}, \rho_{\text{Global}}) \approx 0.76$, *every single-factor marginal* X *(Age, Sex, Smoking, Diabetes, BP, Chol) satisfies* $F((\rho_{\text{India}})_X,\ (\rho_{\text{Global}})_X) \geq 0.76$. *More generally, any subset of factors has fidelity at least* 0.76.

Proof. We already know from Proposition 3 that fidelity is monotone under CPTP maps. Discarding variables is a special case—applying the partial trace $\Phi = \mathrm{Tr}_{\bar{X}}$ is a perfectly valid CPTP map. Using it on both cohort states gives us

$F((\rho_{\text{India}})_X,\ (\rho_{\text{Global}})_X) = F(\mathrm{Tr}_{\bar{X}}(\rho_{\text{India}}),\ \mathrm{Tr}_{\bar{X}}(\rho_{\text{Global}})) \;\; \geq \;\; F(\rho_{\text{India}}, \rho_{\text{Global}})$ ....(S26)

Since we have $F(\rho_{\text{India}}, \rho_{\text{Global}}) \approx 0.76$, the bound tells us directly that

$F((\rho_{\text{India}})_X,\ (\rho_{\text{Global}})_X) \;\; \geq \;\; 0.76$ ....(S27)

And of course, this reasoning applies to any subset of risk factors, not just one variable at a time; any combination of the remaining factors can be traced out and the same lower bound applies.

Corollary S3. *Trace-Distance Bounds from Fidelity Let* $D(\rho,\sigma) := 1/2 \parallel \rho - \sigma \parallel_1$ *be the trace distance. The Fuchs–van de Graaf inequalities yield* $1 - \sqrt{F(\rho,\sigma)} \;\leq\; D(\rho,\sigma) \;\leq\; \sqrt{1 - F(\rho,\sigma)}$. *With* $F(\rho_{\text{India}}, \rho_{\text{Global}}) \approx 0.76$, *we obtain* $0.129 \;\lesssim\; D(\rho_{\text{India}}, \rho_{\text{Global}}) \;\lesssim\; 0.490$. *Thus the cohorts differ by a nontrivial total-variation–like distance, consistent with a* 24% *systemic divergence.*

Proof. The Fuchs–van de Graaf (FvG) inequalities state that for any density operators ρ, σ,

$$1 - \sqrt{F(\rho,\sigma)} \;\leq\; D(\rho,\sigma) \;\leq\; \sqrt{1 - F(\rho,\sigma)},$$

where $F(\rho,\sigma) = (\mathrm{Tr}\sqrt{\sqrt{\rho}\,\sigma\,\sqrt{\rho}})^2 \in [0,1]$ is the Uhlmann fidelity and $D(\rho,\sigma) = 1/2 \parallel \rho - \sigma \parallel_1$.

We apply these two scalar inequalities directly to the reported whole-system fidelity $F(\rho_{\text{India}}, \rho_{\text{Global}}) \approx 0.76$. Since both bounding functions $F \mapsto 1 - \sqrt{F}$ and $F \mapsto \sqrt{1 - F}$ are continuous and monotone on $[0,1]$, substituting $F \approx 0.76$ gives numerical bounds on D:

Lower bound:

$$1 - \sqrt{F} \;\approx\; 1 - \sqrt{0.76} \;=\; 1 - 0.8717797887\ldots$$

$$=\; 0.1282202113\ldots \;\approx\; 0.128.$$

Upper bound:

$$\sqrt{1-F} \;\approx\; \sqrt{1-0.76} \;=\; \sqrt{0.24} \;=\; 0.4898979485\ldots$$
$$\approx\; 0.490.$$

Rounding to three significant digits yields

$$0.129 \;\lesssim\; D(\rho_{\text{India}}, \rho_{\text{Global}}) \;\lesssim\; 0.490,$$

as claimed. The nonzero lower bound shows a strictly positive trace distance (hence statistical distinguishability). The appearance of $1 - F = 0.24$ in the upper bound reflects that for pure states the FvG upper bound is tight, i.e., $D = \sqrt{1-F}$.
